# Dietary patterns associated with anaemia among pregnant women in Suva, Fiji

**DOI:** 10.1186/s12884-026-09613-0

**Published:** 2026-07-06

**Authors:** Arishma A. Prasad, Eunice Okyere, Kissinger Marfoh, Ramneek N. Goundar, Puja Nair, Cameron C. Grant

**Affiliations:** 1 Department of Primary Care and Nutrition, School of Public Health and Primary Care, Fiji National University, Suva, Fiji; 2 Department of Public Health, and Health Services Management, School of Public Health and Primary Care, Fiji National University, Suva, Fiji; 3 Department of Epidemiology and Environmental Health, School of Public Health and Primary Care, Fiji National University, Suva, Fiji; 4 Department of Paediatrics: Child & Youth Health, University of Auckland, Auckland, New Zealand

**Keywords:** Dietary pattern, Nutrients, Anaemia in pregnant, Reduced rank regression

## Abstract

**Background:**

Anaemia is prevalent during pregnancy in Fiji. In many countries, inadequate nutrient intake is associated with anaemia during pregnancy. We aimed to describe the relationship between dietary patterns and anaemia amongst pregnant women in Fiji.

**Methodology:**

A hospital-based, age-matched case-control study was conducted in the Antenatal Clinic at the main hospital in Suva, Fiji. Anaemia was defined as a haemoglobin concentration of less than 11.0 g/dL in trimesters 1 and 3, and less than 10.5 g/dL in trimester 2. Dietary variables were assessed using a semi-quantitative food frequency questionnaire, primarily reflecting late-pregnancy intake. Reduced-Rank Regression was employed to identify dietary patterns. Hierarchical multivariable conditional logistic regression models were used to assess the relationship between dietary patterns and anaemia in pregnancy.

**Results:**

We enrolled 52 cases and 52 controls. The adjusted odds of anaemia decreased among women who were employed (aOR = 0.20, 95% CI: 0.11–0.37), had at least four antenatal visits (aOR = 0.30, 95% CI: 0.01–0.60), or did not drink milk with main meals (aOR = 0.04, 95% CI: 0.01–0.63). As the intake of vitamin B9 (aOR = 0.85, 95% CI: 0.76–0.98), vitamin B12 (aOR = 0.40, 95% CI: 0.17–0.95), or iron (aOR = 0.42, 95% CI: 0.22–0.80) increased, the odds of anaemia decreased. The odds of anaemia were reduced (aOR = 0.35, 95% CI: 0.10–0.96) for women with high intakes of fruit, vegetables, fish, and nuts, along with a low intake of organ meats, and increased (aOR = 1.21, 95% CI: 1.03–4.02) for women with high intakes of fruit but low intakes of green leafy vegetables, nuts, cereals, and animal protein.

**Conclusion:**

In Fiji, both non-dietary and dietary factors are associated with the risk of anaemia during pregnancy. Preventing anaemia in pregnancy should focus on improving employment opportunities, promoting antenatal care visits, and encouraging nutrient-rich dietary habits.

## Background

Pregnancy is a critical life phase during which adequate nutrition is essential to ensure the health and well-being of both the mother and the baby [1, 2]. Poor nutrition during pregnancy has lasting effects on maternal and infant health [3]. Anaemia in pregnancy is a condition characterised by a reduction in red blood cells or haemoglobin [4], and it is one of the most common causes of preventable obstetric complications. Anaemia is associated with adverse maternal outcomes such as death, the need for blood transfusion, post-partum haemorrhage, and reduced quality of life [4]. In addition, it is linked to poor fetal outcomes like preterm birth, small-for-gestational-age infants, and perinatal fetal death [4, 5].

Globally, anaemia affects approximately 50% of pregnancies. According to the World Health Organisation, the prevalence of anaemia among pregnant women aged 15 to 49 years was 36.5% in 2019 [6, 7]. The prevalence is particularly high in the regions of South East Asia (47.8%) and Africa (45.8%) [8]. In the Western Pacific region, anaemia affects an estimated 21.3% of pregnant women [8]. In Fiji, the prevalence of anaemia among pregnant women was 41.4% in 2019, exceeding the WHO’s cut-off, and highlighting it as a significant public health concern [9].

Iron deficiency accounts for 50% of all anaemic cases in pregnancy and is estimated to cause 591,000 perinatal deaths and 115,000 maternal deaths annually [10]. This report shows the critical role of nutritional deficiencies in anaemia during pregnancy. Inadequate intake of essential nutrients, such as iron, folate, vitamin B12, and vitamin A, has been linked to an increased risk of anaemia in pregnancy [9, 11, 12]. Such deficiencies not only heighten the risk of anaemia in pregnancy but also contribute to its persistence as a major public health issue.

Although the relationship between nutrients and anaemia is well-established, the relationships between dietary patterns and anaemia in pregnant women are less understood. Previous studies have primarily focused on obstetric risk factors, while nutrients and diet have received less attention [13–15]. Studies have shown that iron, folate and vitamins help in red blood cell formation in pregnant women [9, 11, 12]. Pregnant women who consume diets rich in red meat, organ meat, and vegetables, - foods that are high in these essential nutrients - have been associated with reduced risk of anaemia and improved pregnancy outcomes [16–19]. However, the extent to which these dietary relationships apply to pregnant women in Fiji remains unexplored, as there is no published data on how these dietary patterns relate to anaemia in this population.

In Fiji, pregnant women attending antenatal clinics are routinely given folate and iron supplements, as well as dietary education, to reduce the risk of anaemia during pregnancy. Despite these measures, the prevalence of anaemia in pregnancy remains high at 41.4% [9], suggesting that cultural dietary habits may influence the risk of anaemia in this population. A better understanding of the relationship between dietary patterns and anaemia in this population could inform interventions designed to reduce the prevalence of anaemia. Therefore, this study aims to investigate the association between dietary patterns and the risk of anaemia among pregnant women attending antenatal care services at the Colonial War Memorial Hospital in Suva, Fiji.

## Method

### Study design

A hospital-based matched case-control study was conducted with a 1:1 case-to-control ratio, with controls matching cases on age.

### Study setting

The study was conducted at the Colonial War Memorial Hospital (CWM) in Suva, Fiji, from December 20, 2022, to March 10, 2023. As the largest healthcare facility in the country, CWM Hospital serves as Fiji’s primary tertiary referral center and the main public hospital for the Central Division. The hospital offers antenatal services to all expectant mothers residing in Lami (peri-urban), as well as in the urban areas of Nasese and Toorak.Furthermore, it receives referrals for pregnant women with health or pregnancy complications from other parts of the Greater Suva Area, such as Raiwaqa, Samabula, and Tamavua.

The CWM serves as both a primary care facility for its immediate vicinity and a tertiary referral centre; therefore, the hospital’s catchment area encompasses a broad and representative socio-economic spectrum. The study population includes individuals ranging from higher-income urban residents to those living in densely populated, lower-income peri-urban settlements. At these clinics, dietary advice is provided as part of a weekly group session led by nurses, a dietitian, and a physiotherapist. These sessions cover topics such as dietary sources of iron, the importance of vitamin C, and inhibitors of iron absorption. Additionally, pregnant women identified as anaemic are offered extra sessions with the dietitian.

### Study population and sample

All pregnant women attending the clinic had their haemoglobin concentrations measured. They were approached and invited to participate in this study on anaemia. The haemoglobin measurements were reviewed for those who provided consent, and a case series of pregnant women with anaemia was thus created. Anaemia was defined as a haemoglobin concentration of < 11.0 g/dl in the first and third trimesters and 10.5 g/dl in the second trimester [20] .

To identify controls, pregnant women of the same age as the case were approached until one was found who was not anaemic, defined as haemoglobin concentration measurements during pregnancy being ≥ 11 g/dl [21].

Pregnant women were ineligible if they had pre-existing medical conditions affecting haemoglobin and diet (such as sickle cell disease, beta thalassemia, chronic kidney disease, diabetes, and hypertension); were diagnosed with anaemia from non-dietary causes (including HIV, malaria, and other acute infections); experienced antepartum haemorrhage due to placenta previa or abruption; had severe pregnancy-related complications like pre-eclampsia or eclampsia; followed restricted diets; or possessed incomplete medical records. The inclusion and exclusion criteria for both cases and controls were identical, except that anaemia was absent in the controls.

### Sample size and power

The sample size was estimated using the two-sample proportional formula [22]. The sample size was estimated to have a statistical power of 80% and an alpha level of 5%. This calculation showed we required a sample size of 104 to detect a clinically significant difference of 20% in the odds of dietary iron intake between the case and control groups [22, 23]. Therefore, we enrolled 52 cases and 52 controls.

### Data collection methods

#### Assessment of diet

A semi-quantitative food frequency questionnaire (FFQ) previously validated by the Food and Agriculture Organization of the United Nations (2018) [24] was used to assess the intake of 31 food items. The FFQ allowed participants to describe the frequency of consumption of specified amounts of each type of food over the previous three months. The FFQ recorded intakes based on the following categories: daily, 3–4 times per week, 1–2 times per week, 1–3 times per month, once a month, and never.

The amount of food consumed was determined by showing pictures of food portions with the amount written, and also through standard household measuring cups, developed by the National Food and Nutrition Centre (Fiji) and a photographic food atlas from Kenya [25, 26]Grams were the unit of measurement. The 31 food items were included because they were consumed in Fiji at the time of the study and were known to be good sources of iron, protein, folate, vitamin B12, vitamin C, and vitamin A.

#### Assessment of daily nutrient intake

The Iron, Protein, Folate, Vitamin B12, Vitamin C, and Vitamin A content of each food item was derived from FoodWorks Nutrient Analysis Software Version 10. The daily nutrient intake was calculated by multiplying the frequency of consumption of each food unit by its nutrient content. The frequency of consumption from the FFQ was converted into daily intake; for example, 1–2 times per week equals 0.21 times per day.

Dietary nutrient intake was then categorized as either adequate or inadequate based on the standard Estimated Average Requirement (EAR) cutoffs for pregnant women. The EAR cutoff values used to define adequacy for protein, vitamin A, vitamin B9 (folate), vitamin B12, vitamin C, and iron were 57 g/day, 550 mcg/day, 520 mcg/day, 2.2 mcg/day, 70 mg/day, and 22 mg/day, respectively.

#### Assessment of non-dietary factors

Socio-demographic status was described using residence, employment status, ethnicity and education. Age was used as the matching variable. Antenatal information included: pregnancy stage, parity, first Antenatal Clinic ANC visit and number of antenatal visits.

### Statistical analysis

Data collected using the questionnaire were coded and transcribed into Microsoft Excel. Data screening was completed to identify any missing data. Analyses were performed using SPSS (Statistical Package for the Social Sciences), except for the reduced rank regression, which was conducted using SAS (Statistical Analysis System) software version 9.4 (SAS Institute, Cary, NC, USA). A p-value of < 0.05 was considered statistically significant.

The distribution of variables describing sociodemographics, pregnancy characteristics, and food patterns was outlined for both cases and controls. Continuous and categorical variables were reported as means and standard deviations, and frequencies and proportions, respectively. Pearson’s correlation coefficient was utilised to assess the relationship between dietary pattern scores and response variables (intake of iron, protein, folate, vitamin B12, vitamin C, and vitamin A). To compare groups, the Student’s T-test was applied for continuous variables, and the Chi-square test was used for categorical variables. Fisher’s exact test was implemented when over 25% of expected cell counts were less than 5.

### Reduced rank regression (RRR) for describing dietary patterns

Dietary patterns were derived using a reduced rank regression method proposed by Hoffmann et al. [26] and evaluated concerning the presence of anaemia during pregnancy. Reduced Rank Regression aims to identify dietary patterns (food combinations) linked to anaemia. In the RRR model, the independent or predictor variables are the food items from the FFQ, and the response variables are the nutrients hypothesised a priori to be associated with anaemia during pregnancy. The model identifies the optimal linear combination of foods that explains the variance in the response variables. For each pregnant woman, a dietary pattern score reflects how closely her diet aligns with a particular dietary pattern. These scores were used to investigate associations between different dietary patterns and anaemia during pregnancy.

### Analysis of dietary patterns and anaemia

Hierarchical multivariable conditional logistic regression models were used to estimate the adjusted odds ratios (OR) and 95% confidence intervals (CI) for the association between dietary factors and anaemia during pregnancy. We fitted five models to identify risk factors linked to anaemia in pregnancy. Model 1 includes sociodemographic factors. Model 2 builds on Model 1 by adding variables that describe pregnancy characteristics. Model 3 adjusts for the variables in Model 2 and includes knowledge and practices related to anaemia. Model 4 adjusts for the variables in Model 3 and includes daily nutrient intake for the six nutrients. Lastly, Model 5 adjusts for the variables in Model 4 and incorporates the six dietary pattern scores. The models were compared against each other using the log-likelihood ratio test (LRT). Multicollinearity was assessed before fitting the final model, and no evidence of problematic multicollinearity was observed.

## Results

### Sociodemographic and antenatal history characteristics

Fifty-two cases (with anaemia) and 52 controls (without anaemia) were identified. The age range for the participants was 18–40 years. A comparison of cases and controls with respect to sociodemographics and pregnancy characteristics showed no significant differences (Table 1).

**Table 1:** Comparison of cases and controls with respect to sociodemographics and pregnancy characteristics showed no significant differences

Variables	Cases (*N* = 52) *n* (column %)*	Control (*N* = 52) *n* (column %)*	*P* value
Socio-demographics Characteristics			
Age in years, mean (s.d.)	28.37 (5.5)	28.94 (6.1)	0.62^†^
Residence			0.39^‡^
Rural	21 (40)	22 (42)	
Urban	31 (60)	30 (58)	
Employment status			0.17^‡^
Not employed	32 (62)	25 (48)	
Employed	20 (38)	27 (52)	
Ethnic group			0.18^§^
i-Taukei	42 (81)	43 (83)	
Fijian-Indian Descent	8 (15)	5 (10)	
Others	2 (4)	4 (7)	
Education			0.56^‡^
Secondary	27 (52)	24 (46)	
Tertiary	25 (48)	28 (54)	
Pregnancy characteristics			
Pregnancy stage			0.70^§^
First trimester	0 (0)	2 (4)	
Second trimester	3 (6)	5 (10)	
Third trimester	49 (94)	45 (86)	
Parity			0.08^ǁ^
Primigravida	10 (19)	18 (35)	
Multipara	42 (81)	34 (65)	
First ANC visit			0.89^§^
Trimester 1	17 (33)	19 (37)	
Trimester 2	27 (52)	22 (42)	
Trimester 3	8 (15)	11 (21)	
No. of Antenatal visits			0.12^‡^
Less than 4 visits	17 (33)	10 (19)	
More than 4 visits	35 (67)	42 (81)	
Foods and drinks consumed with main meal during pregnancy			
Fruit			0.49^ǁ^
Always	29 (56)	32 (63)	
Sometimes	8 (15)	8 (15)	
No	15 (29)	12 (23)	
Black or milk tea			0.23^ǁ^
Always	38 (73)	32 (61)	
Sometimes	2 (4)	3 (6)	
No	12 (23)	17 (33)	
Coffee			0.07^ǁ^
Always	2 (4)	7 (13)	
Sometimes	4 (8)	5 (10)	
No	46 (88)	40 (77)	
Milk			0.91^ǁ^
Always	34 (65)	34 (65)	
Sometimes	4 (8)	3 (6)	
No	14 (27)	15 (28)	

* n (column %) unless otherwise stated, † t-test, ‡ χ_1_^2^, § Fisher exact test, ǁ χ^2^_trend_

### Consumption of fruit, milk, coffee and tea with the main meal

The proportions of cases and controls not consuming fruit (cases 29%, controls 23%, *p* = 0.49), those always or sometimes consuming milk (cases 73%, controls 71%, *p* = 0.90), and tea (cases 77%, controls 67%, *p* = 0.23) with the main meal did not differ.

### Daily nutritional uptake among pregnant women

Table 2 shows the association between dietary nutrient inadequacy and the odds of anaemia. Generally, inadequate nutrient intake was more prevalent among cases than controls. Analysis revealed that pregnant women with inadequate dietary iron had 3.60-fold higher odds of anaemia compared with those with adequate intake (95% CI: 1.55–8.37). Although inadequate intakes of vitamin B12 (OR = 1.94; 95% CI: 0.78–4.94) and folate (OR = 1.83; 95% CI: 0.69–4.89) were more common among cases, they were not significantly associated with increased odds of anaemia. Similarly, no significant associations were observed for dietary protein, vitamin A, or vitamin C.

**Table 2:** Comparison of dietary nutrient adequacy between cases and controls, and the associated odds of anaemia

Nutrient Intake	Cases *n* (%)	Controls *n* (%)	OR (95% CI)
Protein			
Adequate	26 (46.4%)	30 (53.6%)	1.00 (Reference)
Inadequate	26 (54.2%)	22 (45.8%)	1.36 (0.63–2.96)
Vitamin A			
Adequate	44 (48.4%)	47 (51.6%)	1.00 (Reference)
Inadequate	8 (61.5%)	5 (38.5%)	1.71 (0.52–5.62)
Folate (Vitamin B9)			
Adequate	39 (47.0%)	44 (53.0%)	1.00 (Reference)
Inadequate	13 (61.9%)	8 (38.1%)	1.83 (0.69–4.89)
Vitamin B12			
Adequate	37 (46.3%)	43 (53.8%)	1.00 (Reference)
Inadequate	15 (62.5%)	8 (37.5%)	1.94 (0.78–4.94)
Vitamin C			
Adequate	42 (49.4%)	43 (50.6%)	1.00 (Reference)
Inadequate	10 (52.6%)	9 (47.4%)	1.14 (0.42–3.08)
Iron			
Adequate	12 (30.8%)	27 (69.2%)	1.00 (Reference)
Inadequate	40 (61.5%)	25 (38.5%)	3.60 (1.55–8.37)*

*P-value > 0.05

### Dietary patterns among pregnant women

Reduced Rank Regression was used to identify six dietary patterns among the study population. The factor loadings for each dietary pattern are presented in Table 3. Factor loadings above 0.2 indicate a high intake of specific food items, while factor loadings below − 0.2 signify a low intake. Six dietary patterns were identified: (1) high intake of fruits, vegetables, and animal protein; (2) high intake of fruits, vegetables, fish, and nuts, along with a low intake of organ meats; (3) high intake of fruits, fish, nuts, and animal protein, and a low intake of vegetables; (4) high intake of fruit and a low intake of vegetables, nuts, cereals, and animal protein; (5) high intake of fruits, nuts, and animal protein, accompanied by a low intake of vegetables and organ meat; (6) high intake of fruits, vegetables, nuts, and cereals. The factor loadings of each food item associated with the six dietary patterns are shown in Table 3.

**Table 3:** Factor loadings for consumption of each of the foods with each of the six dietary patterns for cases and controls

Food Group and Item	Factor Loadings* for each dietary pattern					
	1^†^	2^‡^	3^§^	4^ǁ^	5^¶^	6^**^
Fruits						
Pawpaw	0.36	0.35		0.41		
Banana	0.24				0.23	
Mango		0.23			0.29	-0.21
Grapes		0.21				-0.21
Pineapple			0.26			
Watermelon					0.30	-0.22
Orange					0.22	-0.33
Pear						-0.29
Apple						-0.36
Vegetables						
Dalo leaves	0.29	0.29	-0.22	-0.41		
Bele	0.26			-0.32		
Spinach	0.22		-0.32	-0.34		
Okra	0.20		-0.22			-0.24
Fern		0.21		-0.29		
Cabbage					0.21	-0.31
Long bean						-0.24
Bokchoy						
Bitter gourd						
Moringa leaves					-0.29	
Meats and Poultry						
Chicken	0.20		0.32	-0.23	0.28	
Fish		0.25	0.53			
Eggs			0.23			
Lamb			0.21	-0.24	0.21	
Beef				-0.20		
Organ Meats						
Giblet	0.43	-0.28			-0.28	
Liver	0.37	-0.49			0.25	
Nuts and Seeds						
Peanuts		0.21		-0.21	0.31	
Almonds					0.24	-0.20
Cashews						
Cereals						
Oats						
Weetbix						

* Factors loading ≥ 0.20 indicate a high intake and factor loading of ≤-0.20 indicates a low intake of the food item within a dietary pattern

† Dietary pattern 1: High intake of fruits, vegetables and meats/organs (Loadings ≥ 0.20 for 2 fruits, 4 vegetables, 1 poultry, and 2 organ meats)

‡ Dietary pattern 2: High intake of fruit, vegetables, fish and nuts, with low intake of organ meats (Loadings ≥ 0.20 for 3 fruits, 2 vegetables, fish, and peanuts; Loadings ≤-0.20 for 2 organ meats)

§ Dietary pattern 3: High intake of fruits, fish, nuts and animal protein, with low intake of vegetables (Loadings ≥ 0.20 for 4 meats/poultry/eggs, fish, 1 fruit, and 1 nut; Loadings ≤ -0.20 for 3 vegetables)

ǁ Dietary pattern 4: High intake of fruit with low intake of vegetables, meats, and nuts (Loading ≥ 0.20 for 1 fruit [pawpaw]; Loadings ≤ -0.20 for 4 vegetables, 3 meats/poultry, and 1 nut)

¶ Dietary pattern 5: High intake of fruits, nuts, and meats, with low intake of specific vegetables and organs (Loadings ≥ 0.20 for 4 fruits, 2 nuts, 2 meats/poultry, 1 organ meat, and 1 vegetable; Loadings ≤- 0.20 for 1 vegetable and 1 organ meat)

** Dietary pattern 6: Low intake of fruits, vegetables, nuts, and cereals (Loadings ≤- 0.20 for 5 fruits, 3 vegetables, 1 nut, and 1 cereal; No positive loadings ≥ 0.20

### Association between dietary patterns, risk factors and anaemia

A hierarchical multivariable conditional logistic regression model was fitted to identify independent associations with the presence of anaemia (Table 4). The full model (model 5) included variables describing sociodemographics, pregnancy characteristics, dietary practices relevant to iron absorption, the intake of six specific nutrients during pregnancy, and six dietary patterns. Variables independently associated with anaemia in pregnancy were employment status, antenatal visits, milk consumption with the main meal, intake of vitamin B9, vitamin B12, iron, and dietary patterns 2 and 4.

**Table 4:** Hierarchical multivariable conditional logistic regression model analysis of risk factors for anaemia

Variables	Model 1	Model 2	Model 3	Model 4	Model 5
	OR (95%CI)	OR [95%CI]	OR [95%CI]	OR [95%CI]	OR [95%CI]
Sociodemographics					
Employment Status					
Not employed	1.00	1.00	1.00	1.00	1.00
Employed	0.61 (0.27–1.4)	0.47 [0.56–1.85]	0.34 [0.11–1.08]	0.17 [0.03–0.91]^†^	0.20 [0.11–0.37]^†^
Educational Level					
Secondary	1.00	1.00	1.00	1.00	1.00
Tertiary	0.99 (0.43–2.32)	1.46 [0.18–1.26]	1.42 [0.53–1.82]	1.97 [0.48–8.13]	1.99 [0.48–8.25]
Residence					
Rural	1.00	1.00	1.00	1.00	1.00
Urban	1.12 (0.53–2.34)	1.07 [0.48–1.40]	1.11 [0.48–1.58]	1.09 [0.32–3.72]	1.07 [0.31–3.72]
Pregnancy characteristics					
Parity					
Primigravida		1.00	1.00	1.00	1.00
Multipara		3.02 [0.95–1.61]	2.95 [0.82–1.63]	1.08 [0.18–6.47]	1.05 [0.16–6.72]
No. of Antenatal visits					
Less than 4 visits		1.00	1.00	1.00	1.00
4 or more visits		0.39 [0.14–1.10]	0.28 [0.08–1.00]	0.29 [0.02–0.86]^†^	0.30 [0.01–0.60]^†^
First ANC visit					
Trimester 1		1.00	1.00	1.00	1.00
Trimester 2 and 3		0.70 [0.27–1.82]	0.73 [0.26–1.06]	0.30 [0.06–1.50]	0.30 [0.06–1.54]
Knowledge Level					
Less knowledgeable			1.00	1.00	1.00
Knowledgeable			2.15 [0.68–1.75]	0.55 [0.12–2.61]	0.54 [0.11–2.61]
Dietary practices relevant to iron absorption					
Do you eat fruits with your main meals?					
Yes*			1.00	1.00	1.00
No			0.72 [0.23–1.18]	1.47 [0.24–9.04]	1.50 [0.24–9.46]
Do you drink black tea or milk tea with main meals?					
Yes*			1.00	1.00	1.00
No			1.17 [0.41–1.34]	0.42 [0.06–2.87]	0.42 [0.06–2.85]
Do you drink milk with main meals?					
Yes*			1.00	1.00	1.00
No			0.50 [0.17–1.48]	0.04 [0.00–0.54]	0.04 [0.01–0.63]^†^
Intake of the six selected nutrients during pregnancy					
Protein (g/day) per 1 standard deviation (36.7) increase				1.01 [1.00–1.01]	1.01 [1.00–1.03]
Vitamin B 9 (mcg/day) per 1 standard deviation (509.4) increase				0.90 [0.74–0.98]^†^	0.85 [0.76–0.98]^†^
Vitamin B 12 (mcg/day) per 1 standard deviation (5.6) increase				0.78 [0.57–0.87]^†^	0.40 [0.17–0.95]^†^
Vitamin C (mg /day) per 1 standard deviation (208.4) increase				1.11 [0.02–2.01]	1.12 [0.01–2.04]
Iron (mg/day) per 1 standard deviation (9.6) increase				0.67 [0.49–0.90] †	0.42 [0.22–0.80]^†^
Vitamin A (mcg RAE/ day) per 1 standard deviation (2520.2) increase				1.00 [1.00–1.00]	1.00 [1.00–1.00]
Dietary Patterns					
Dietary Pattern 1					0.93 [0.63–1.37]
Dietary Pattern 2					0.35 [0.10–0.96]^†^
Dietary Pattern 3					1.28 [0.75–1.20]
Dietary Pattern 4					1.21 [1.03–4.02]^†^
Dietary Pattern 5					1.27 [0.71–1.27]
Dietary Pattern 6					1.53 [0.14–14.01]
Model Fit Statistics					
** -**2Log-likelihood (degrees of freedom)	81.3 (3)	74.4 (6)	68.9 (10)	55.4 (16)	37.2 (22)
LRT p-value (Change from previous model)	0.539	0.075	0.240	0.036	0.006

Dietary pattern 1: High intake of fruits, vegetables and animal protein.Dietary pattern 2: High intake of fruit, vegetables, fish and nuts and low intake of organ meats.Dietary pattern 3: High intake of fruits, fish, nuts, animal protein, and low intake of vegetables.Dietary pattern 4: High intake of fruit and low intake of green leafy vegetables, nuts, cereals and animal protein.Dietary pattern 5: High intake of fruits, nuts and animal protein and low intake of vegetables and organ meat.Dietary pattern 6: Low intake of fruits, vegetables, nuts and cereals.

* For the multiple variable analysis, ‘yes’ and ‘sometimes’ combined and compared with ‘no’ **†** Statistically significant finding (*p* < 0.05).

The odds of anaemia in pregnancy decreased in employed women compared to those unemployed (OR = 0.20; 95% CI: 0.11–0.37). Pregnant women with four or more antenatal visits had a 70% reduction in the odds of anaemia compared to those with fewer than four antenatal visits (OR = 0.30; 95% CI: 0.01–0.60). Conversely, pregnant women who did not consume milk with main meals had significantly lower odds of anaemia compared to those who did (OR = 0.04; 95% CI: 0.01–0.63). Furthermore, increased dietary intake of vitamin B9 (OR = 0.85; 95% CI: 0.76–0.98), vitamin B12 (OR = 0.40; 95% CI: 0.17–0.95), and iron (OR = 0.42; 95% CI: 0.22–0.80) provided protection against anaemia in pregnancy (Table 4).

Using the dietary patterns derived from the reduced rank regression analysis, we found that pregnant women with diets high in fruit, vegetables, fish, and nuts, and low in organ meats (Dietary Pattern 2) had a 65% decrease in the odds of anaemia (OR = 0.35; 95% CI: 0.10–0.96). However, pregnant women with diets high in fruit but low in green leafy vegetables, nuts, cereals, and animal protein (Dietary Pattern 4) showed a significant 21% increase in the odds of anaemia (OR = 1.21; 95% CI: 1.03–4.02) (Table 4).

## Discussion

This study aimed to identify risk factors associated with anaemia during pregnancy, focusing on dietary habits. It assessed various factors, including socio-demographic characteristics, antenatal history, knowledge and practices, and dietary patterns. A hierarchical multivariable conditional logistic regression model was employed to understand the collective effects of these factors on anaemia in pregnancy.

Visiting the antenatal clinic four or more times was associated with a 70% decrease in the odds of anaemia. In studies conducted in low- and middle-income countries across Asia, Africa, Latin America and the Caribbean, antenatal care attendance has been shown to be associated with better adherence to iron supplementation recommendations [27, 28]. Antenatal clinics are an important source of information on health and nutrition, and they serve as the primary distribution point for routine prenatal supplements. Thus, increasing antenatal visits to at least four times is a critical maternal health intervention to prevent anaemia. Although compliance with iron-folic acid supplementation was not directly measured in this study, frequent antenatal attendance may have increased women’s opportunities to receive supplements, counselling, and reinforcement of prescribed supplementation [29].

We found that employed pregnant women had significantly lower odds of anaemia than those who were unemployed after controlling for other variables. Employment is linked to reduced odds of anaemia in similar studies conducted in Bangladesh, Ethiopia, and Nigeria [30–32]. In the present study, this association may reflect better purchasing power and access to nutrient-rich foods among employed women. Employment generally provides a regular income, which may offer the financial stability needed to support a healthier diet. The ability to purchase nutrient-rich foods, particularly animal-source proteins and fresh produce, may contribute to better dietary quality and reduce the risk of anaemia in pregnancy. Furthermore, employed women may have better access to healthcare services, either through workplace programmes or the financial means to afford private healthcare, facilitating earlier detection and treatment of anaemia.

Regarding dietary practices, consuming milk with main meals was associated with increased odds of anaemia, whereas those who did not drink milk with meals had lower odds of anaemia (OR = 0.04; 95% CI: 0.01–0.63). Research indicates that the efficiency of iron absorption can decrease when calcium-rich foods are consumed simultaneously with iron sources [33]. When milk is consumed with main meals, the calcium may reduce non-haem iron absorption, particularly when the meal is mainly plant-based or contains other inhibitors of iron absorption such as phytates and polyphenols. This physiological mechanism is well-recognized in nutritional studies [34–37], supporting the separation of dairy and iron-rich meals as a practical dietary recommendation during pregnancy.

We compared the intake of six selected nutrients (protein, vitamin B9, vitamin B12, vitamin C, iron, and vitamin A) to the Estimated Average Requirement (EAR) cut-offs for pregnancy. Our results showed that inadequate iron intake was the only nutrient significantly associated with higher odds of anaemia in the unadjusted analysis (OR = 3.60; 95% CI: 1.55–8.37). Although inadequate intakes of folate, vitamin B12, protein, vitamin A, and vitamin C were also more common among pregnant women with anaemia, these associations did not reach statistical significance. While EAR-based studies have shown that inadequate dietary iron intake is common during pregnancy, direct comparisons are limited because many of these studies did not assess anaemia as an outcome [38, 39].

When nutrient intakes were analysed as continuous variables in the fully adjusted hierarchical conditional logistic regression model, higher intakes of iron, vitamin B9, and vitamin B12 were significantly associated with lower odds of anaemia. In contrast, protein, vitamin C, and vitamin A intakes were not significantly associated with anaemia. This finding is consistent with the biological role of iron, folate, and vitamin B12 in haemoglobin synthesis and red blood cell production. Studies from India and the United States have similarly reported that higher iron intake is associated with reduced odds of anaemia during pregnancy [40, 41]. A study in Senegal also shown a protective association between vitamin B9 intake and anaemia during pregnancy [42], while vitamin B12 deficiency is a recognised contributor to anaemia in pregnant women [43–45]. Overall, the findings from both the EAR-based and continuous nutrient-intake analyses point to iron as the most consistent nutrient associated with anaemia, with vitamin B9 and vitamin B12 also contributing to the risk of anaemia when intake was assessed as a continuous variable in the hierarchical model.

Importantly, the dietary assessment was performed during the third trimester for the vast majority of pregnant women. This timing is important for interpreting the nutrient findings because the physiological demand for iron is exceptionally high during this period and may be difficult to meet through diet alone, particularly among pregnant women who habitually consume plant-based diets. Therefore, the observed association between dietary iron inadequacy and anaemia should be interpreted in the context of both habitual food intake and routine iron-folic acid supplementation during pregnancy. Our finding that dietary iron inadequacy was associated with increased odds of anaemia supports the importance of adequate iron intake during pregnancy and is consistent with WHO recommendations on routine iron-folic acid supplementation [29]. The importance of adequate iron intake and supplementation is further supported by previous studies from Pakistan, Egypt, Somalia, and China, where inadequate adherence to supplementation has been associated with anaemia among pregnant women [39, 41, 46–48]. Therefore, dietary counselling should be considered complementary to routine iron-folic acid supplementation, particularly in the second and third trimesters when iron requirements are highest.

We acknowledge that the association between a single nutrient and anaemia is difficult to assess using dietary intake data alone. This is because nutrients are consumed as part of foods, composite meals, and dietary patterns rather than in isolation [36, 49–51]. The effect of individual nutrient intakes can be modified by the food matrix, including the physical structure of foods, the chemical form of nutrients, and the presence of absorption inhibitors and enhancers [36, 49]. Furthermore, in a population of pregnant women, the observed association between dietary nutrients and anaemia may also be influenced by the consumption of prescribed multiple micronutrients.For this reason, the dietary pattern analysis used in this study allows the simultaneous evaluation of correlated dietary constituents, rather than assessing each nutrient separately [36, 51].

Reduced Rank Regression was employed to derive six dietary patterns, as no single nutrient is consumed in isolation [36, 49–51]. This approach to deriving dietary patterns differs from Principal Component Analysis and Factor Analysis, which are primarily data-driven methods based on correlations among food groups. In contrast, Reduced Rank Regression integrates a priori disease-related nutrient information through selected response variables, thereby linking dietary patterns more directly to the outcome of interest [52]. Therefore, RRR provides a useful way to evaluate the interrelationships of dietary variables and their combined association with anaemia. This aids in exploring the complex relationships between diet and disease while accounting for the cumulative and interactive effects of various dietary components [36, 49–51].

Among the six derived dietary patterns, we identified two that were significantly related to anaemia in pregnancy. Dietary Pattern 2, characterised by a high intake of fruit, vegetables, fish, and nuts, was significantly associated with a 65% reduction in the odds of anaemia (OR = 0.35; 95% CI: 0.10–0.96). Conversely, Dietary Pattern 4, characterized by a high intake of fruit but a low intake of green leafy vegetables, nuts, cereals, and animal protein, was significantly associated with 21% with increased in odds of aneamia (OR = 1.21; 95% CI: 1.03–4.02). These dietary patterns may help explain the observed association between diet and anaemia. Animal food sources (such as fish and meats), provide highly bioavailable haem iron [36, 37, 53]. Plant sources, such as dark green leafy vegetables, including dalo leaves, and nuts, provide non-haem iron and folate (vitamin B9) [36, 37, 53]. However, the absorption of non-haem iron is enhanced by the co-consumption of vitamin C (ascorbic acid), while phytates, polyphenols, calcium, and some dietary proteins may reduce iron bioavailability [37, 54]. Therefore, Dietary Pattern 2 may be protective because it combines sources of non-haem iron (vegetables and nuts) and haem iron (fish) with fruits rich in vitamin C (such as pawpaw and citrus), which enhances non-haem iron absorption. In contrast, Dietary Pattern 4 may increase the risk of anaemia because it is low in green leafy vegetables, nuts, cereals, and animal-source foods, which are important dietary sources of iron and other nutrients required for haemoglobin synthesis. Although this pattern includes vitamin C from fruits, the lack of sufficient iron-rich foods may limit its protective effect. These pattern-level findings are consistent with the nutrient-level result showing that inadequate iron intake was the main dietary factor associated with anaemia in this study.

This study utilised a case-control design with a precise case definition and age-matched controls, whom we confirmed did not meet the case definition. A wide range of potential determinants of anaemia was measured, including socio-demographic characteristics, antenatal history, and dietary patterns, as well as dietary consumption and quality. We considered a comprehensive variety of variables associated with the risk of anaemia. The dietary pattern analysis allowed for the simultaneous evaluation of correlated dietary constituents, which is important because foods and nutrients are commonly consumed together. The hierarchical multivariable conditional logistic regression model enabled adjustment for potentially confounding factors and the identification of the independent association of each variable with anaemia risk.

The limitations of the study include, as with any observational study, the inability to demonstrate causality due to potential residual confounding. One limitation is that diet was measured only at one point during the pregnancy using a Food Frequency Questionnaire (FFQ), which assessed average dietary intake over three months. Most women (94% of cases, 86% of controls) were interviewed during the third trimester. While the potential for recall bias exists, questioning women about their diet prior to childbirth minimises the bias created by drastic dietary changes that occur post-partum. The study utilised a semi-quantitative FFQ, which relies on dietary assessment techniques commonly used to capture habitual dietary intake in population-based nutrition studies [24]. However, FFQs are population-specific, and the food list, portion sizes, and nutrient estimates depend on local dietary habits and the objectives of the study. Therefore, recall bias, portion-size estimation error, and misclassification of usual intake are particularly problematic when dietary intake changes drastically during pregnancy. Another limitation was that the study did not capture specific cooking methods, such as boiling, frying, or prolonged cooking, which may affect nutrient retention and bioavailability. Although antenatal attendance may have served as a proxy for opportunities to receive iron-folic acid supplementation, adherence to supplementation was not directly measured. This may have influenced the observed associations between antenatal attendance, dietary intake, and anaemia. Finally, the generalizability of the results may be limited due to the specific demographic and cultural characteristics of our study population in Fiji. Therefore, the results may be most applicable to pregnant women attending antenatal care in similar clinical and dietary settings.

## Conclusion

Anaemia in pregnancy remains a significant public health issue in Fiji. This study underscored the relevance of both dietary and non-dietary factors as independent predictors of the risk of anaemia in pregnant women. These results highlight the need for comprehensive anaemia prevention strategies that extend beyond individual nutrient recommendations. Effective interventions must address socioeconomic barriers, promote routine antenatal care attendance, and encourage nutrient-rich dietary patterns that maximize iron bioavailability to promote overall maternal health.

## Data Availability

The datasets used for this study are available from the corresponding author on reasonable request.
